# Acute Shoulder Monoarthritis in a Patient With Acute Myelomonocytic Leukemia With Novel Translocation t(5;13)

**DOI:** 10.4021/wjon2010.02.194w

**Published:** 2010-02-01

**Authors:** Michelle Mueller, Alejandro R. Calvo

**Affiliations:** a Kettering Medical Center, Internal Medicine Residency Program, Department of Medical Education, Dayton, OH, USA; b Sycamore Hospital, Department of Medical Oncology and Hematology, Dayton, OH, USA

**Keywords:** Acute myelogenous leukemia, t(5, 13), Arthritis, Hypercalcemia

## Abstract

We present the case of a patient with acute myelomonocytic leukemia with trisomy 8 and novel translocation t(5;13). In addition to acute leukemia she had debilitating left shoulder arthritis due to granulocytic sarcoma formation in the joint space. Her shoulder pain did not improve during induction chemotherapy but she experienced rapid relief of symptoms with use of local radiation. Her leukemia was found to be primary refractory to chemotherapy and despite an attempt at salvage therapy she died 2 months after diagnosis.

## Case Report

**Figure 1:**
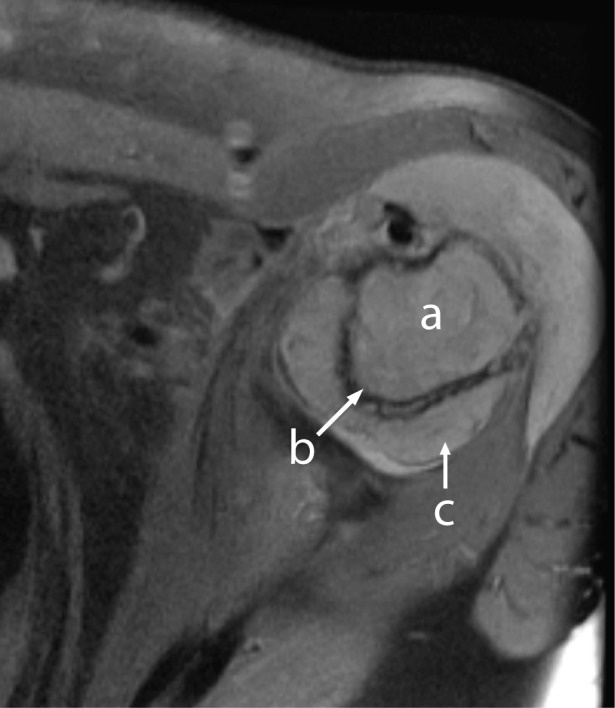
MRI of left shoulder showing abnormal marrow signal of the proximal humerus (a) with cortical erosion (b) and soft tissue mass (c) with joint effusion.

A 60-year-old previously healthy female presented with a 3-week history of increasing left shoulder pain; one week of nausea, abdominal pain, fevers, chills, and new-onset confusion. Laboratory evaluation demonstrated a white blood cell count of 22,600 per cubic millimeter with 28% blasts, hematocrit of 44%, platelet count of 102,000 per cubic millimeter, and calcium of 15.2 mg/dL. AML subtype M4 (according to the French-American-British classification) was confirmed by bone marrow biopsy. Cytogenetics showed trisomy 8 and t(5;13). Bone scan and bone survey were unremarkable. The patient was given standard induction therapy with cytarabine and idarubicin. Zolendronic acid was given for hypercalcemia. Left shoulder pain continued to worsen. Day 14 bone marrow biopsy showed persistent blasts; thus salvage therapy with mitroxantrone and etoposide was initiated. Shoulder pain improved partially, MRI of the left shoulder was performed showing a soft tissue mass in the glenohumeral joint space with cortical erosion of the proximal humerus (Fig 1). Biopsy was consistent with granulocytic sarcoma related to AML with infiltration into the bone and joint space, accounting for findings of hypercalcemia. Pain improved with chemotherapy and local radiation. Hypercalcemia responded to bisphosphonates. The patient had a transient remission, but soon relapsed and died from sepsis.

## Discussion

This case presents three unusual findings; granulocytic sarcoma within the joint space, a translocation not previously reported in AML, and hypercalcemia as a presenting sign of acute myelomonocytic leukemia.

Granulocytic sarcoma is a collection of granulocytic precursor cells, named chloroma for its greenish appearance under the skin due to presence of myeloperoxidase. It is usually associated with AML, particularly subtypes 4 and 5, or the blastic phase of CML. Granulocytic sarcoma may present anywhere in the body, but most commonly appears in the skin, soft tissue, lymph nodes and bone [1]. When found in the bone, it is thought that the cells divide in the bone marrow and travel through the haversian canals to the subperiosteum causing a boney lesion [2, 3]. There can be, however, a nodular appearance of the bone marrow in area of the epiphysis that can be confused with granulocytic sarcoma in AML patients, thus biopsy would be necessary for diagnosis [4]. In the case of our patient the cells actually disrupted the cortex, collecting in the joint space as a solid mass.

Leukemic arthritis is typically pauciarticular involving large joints, but may also present as a polyarthritis similar to rheumatoid arthritis [5, 6]. Leukemic patients can obviously suffer from crystalline, septic and hemorrhagic arthritis; but leukemic cells may also play a direct role in the arthritis. In some patients leukemic infiltration may be seen on synovial tissue biopsy; however, this is unreliable due to patchy involvement of the synovium [7]. In fact several studies have shown no abnormalities in the synovial biopsy specimens of leukemic patients with the use of light, immunofluorescent and electron microscopy where crystalline disease had been excluded [8, 9]. Some patients will have cells in the fluid identifiable through immunocytological analysis or even light microscopy [10, 11].

Review of literature shows very few cases of granulocytic sarcoma in the joint space. One case involved the glenohumeral joint in a patient with essential thrombocytosis, and another case involved the hip joint in a patient with acute promyelocytic leukemia [12, 13]. Additionally, a case of sacroilitis was described in a patient with secondary AML following myelodysplastic syndrome; however, it appears that this was a paraneoplastic phenomenon [14].

Our patient had poor prognostic cytogenetics with trisomy 8 as well as t(5;13), the latter not previously described in AML. Trisomy 8 in the setting of AML has been suggested to be associated with leukemia cutis, the skin infiltration of granulocytic sarcoma [15]. Our patient had a very aggressive leukemia with granulocytic sarcoma in the joint space along with leukemia cutis at the end of her clinical course. The presence of t(5;13) in AML and its relation with refractory disease, hypercalcemia of malignancy and granulocytic sarcoma remains to be defined. 
